# Fault Diagnosis of Hydraulic Components Based on Multi-Sensor Information Fusion Using Improved TSO-CNN-BiLSTM

**DOI:** 10.3390/s24082661

**Published:** 2024-04-22

**Authors:** Da Zhang, Kun Zheng, Fuqi Liu, Beili Li

**Affiliations:** College of Automation and Electronic Engineering, Qingdao University of Science and Technology, Qingdao 266061, China; zk@mails.qust.edu.cn (K.Z.); qdlfq1@163.com (F.L.); nigcmlfy@163.com (B.L.)

**Keywords:** hydraulic system, fault diagnosis, information fusion, convolutional neural network, bi-directional long short-term memory network, tuna swarm optimization

## Abstract

In order to realize the accurate and reliable fault diagnosis of hydraulic systems, a diagnostic model based on improved tuna swarm optimization (ITSO), optimized convolutional neural networks (CNNs), and bi-directional long short-term memory (BiLSTM) networks is proposed. Firstly, sensor selection is implemented using the random forest algorithm to select useful signals from six kinds of physical or virtual sensors including pressure, temperature, flow rate, vibration, motor power, and motor efficiency coefficient. After that, fused features are extracted by CNN, and then, BiLSTM is applied to learn the forward and backward information contained in the data. The ITSO algorithm is adopted to adaptively optimize the learning rate, regularization coefficient, and node number to obtain the optimal CNN-BiLSTM network. Improved Chebyshev chaotic mapping and the nonlinear reduction strategy are adopted to improve population initialization and individual position updating, further promoting the optimization effect of TSO. The experimental results show that the proposed method can automatically extract fusion features and effectively utilize multi-sensor information. The diagnostic accuracies of the plunger pump, cooler, throttle valve, and accumulator are 99.07%, 99.4%, 98.81%, and 98.51%, respectively. The diagnostic results of noisy data with 0 dB, 5 dB, and 10 dB signal-to-noise ratios (SNRs) show that the ITSO-CNN-BiLSTM model has good robustness to noise interference.

## 1. Introduction

Hydraulic systems are widely used in engineering machinery, metallurgy, mining and other fields because of their fast response, strong load resistance, small size, large driving force, and wide speed range. Component and hydraulic oil in a hydraulic system work in a closed oil circuit, and it is difficult to troubleshoot [1]. The failure of the hydraulic system leads to the decline in product quality or the interruption of production activities. Serious accidents such as hydraulic cylinder explosions and hydraulic oil pipe bursts may occur. As important parts of the hydraulic system, the working states of the plunger pump, cooler, throttle valve, and accumulator directly affect the state of the system. By monitoring the flow, pressure, temperature, and other parameters of the hydraulic system, the working status of the components can be recognized and fault information can be provided to maintenance personnel in a timely manner. Condition-Based Maintenance (CBM) not only reduces waste caused by Time-Based Maintenance (TBM) but also maximizes the prevention of serious faults. Due to the interference of complex conditions such as the operation of other parts and external vibration, the extraction of fault features in the signals collected by the sensor becomes very complicated. The timely and accurate fault diagnosis of hydraulic components is of great significance to ensure the safe and stable operation of hydraulic systems [2,3,4].

With the wide application of hydraulic systems, the requirements for their safety and reliability are getting higher and higher, and the fault diagnosis technology of hydraulic systems is getting more and more attention. The methods of fault diagnosis are mainly divided into three main categories: methods based on machine learning and artificial intelligence, methods based on physical models, and methods based on signal processing. X. Peng et al. [5] addressed the importance of the inability of deep learning algorithms to discriminate multi-channel features and built a multi-channel data-driven framework based on a deep residual network (ResNet) and an Attention Mechanism for the fault diagnosis of hydraulic systems. Hanlin Guan et al. [6] used an improved Squeeze-and-Excitation network to redistribute the weights of the fault features in order to solve the problem of the difficult fault detection of hydraulic multi-way valves; the gated recurrent unit (GRU) extracted the features and fused them, and then performed the diagnosis, which improved the stability and diagnostic accuracy compared to the traditional method. D.-N. Chen et al. [7] proposed a multi-sensor information fusion fault diagnosis method combining improved wavelet packet decomposition (WPD) as well as a kernelized support tensor trainer (KSTTM), which effectively improved the classification accuracy and was robust to problems regarding small sample size. Chenzhao Bai et al. [8] designed a high-quality sensor to detect the pollution of hydraulic oil. This model greatly reduced the detection time and improved the detection efficiency. Zhiwei Qiu et al. [9] used a physical model-driven method to realize the intelligent fault diagnosis of the hydraulic system of the shield machine and compared it with the traditional diagnosis method to prove the superiority of the proposed method. Zechao Wang et al. [10] proposed a two-stage model-driven method to compensate for dynamic pressure measurement, which was used in an industrial hydraulic pipeline monitoring system. The compensation measures effectively reduced the error. Jinchuan Shi et al. [11] improved the accuracy of fault diagnosis by converting multi-sensor heterogeneous data into image features and performing feature fusion. Zhiwei Qiu et al. [12] used wavelet packet transform to extract energy features and applied the fusion features to the fault diagnosis of hydraulic cylinders. Changpu Yang et al. [13] studied the beneficial part of noise to intelligent fault diagnosis and improved the accuracy of fault classification by injecting different degrees of noise into the model. Minghang Zhao et al. [3] proposed a new vibration data enhancement method to balance health and fault samples, so as to improve the accuracy and performance of fault diagnosis. Myeong-Seok Lee et al. [14] combined artificial intelligence and physical modeling to analyze the health status of hydraulic gear pumps. M. Wang et al. [15] proposed a classification method (LS-TF) based on a hybrid model of transformer and long- and short-term memory (LSTM) neural networks. Shi Li [16] applied convolutional neural networks (CNNs) for the fault diagnosis of industrial fans’ centrifugal pumps. Yujia Liu et al. [17] proposed a CNN-LSTM fault diagnosis method for chain jacks.

In this paper, a CNN is adopted to extract the deep representation of multi-sensor data, and a BiLSTM network is applied to analyze the forward and backward data features contained in the time series data (the ascending order and the descending order of the time axis). And then, the fault diagnosis of hydraulic components is realized. The model parameter setting has a great influence on the network performance and the diagnostic accuracy [18,19,20]. We aim to address the problems of insufficient search accuracy and the imbalance between global searches and local explorations in the tuna swarm optimization algorithm (TSO) when solving high-dimensional complex optimization problems. In this paper, an improved tuna swarm optimization algorithm (ITSO) based on the combination of Chebyshev chaotic mapping and a nonlinear strategy is proposed to diversify the population and enhance the search capability. The ITSO algorithm is utilized to optimize the learning rate, regularization coefficient, and number of nodes of the BiLSTM layer, which solves the local optimum and achieves good diagnostic results. In view of the large number of sensors in the hydraulic system, including pressure, temperature, flow, and other signals, a single feature cannot accurately and effectively identify the fault state. In this paper, the data collected by multi-sensors are fused at the feature level, and the multi-source data are comprehensively utilized to accurately describe the fault state of hydraulic components. This paper presents the following innovations:

1. The feature-level fusion of multi-sensor information fully utilizes the data from six kinds of sensors and improves the accuracy and robustness of fault diagnosis.
2. Combining a CNN with BiLSTM, using the sparse connection and weight sharing characteristics of CNNs, the learning ability of the network to fault data is improved, and the feature information contained in the data is fully extracted.
3. An ITSO algorithm is proposed. Improved Chebyshev chaotic mapping is adopted to replace the random sequence of the standard TSO initialization, which increases the population diversity and improves the stability of global searches and local explorations. The weight coefficients of the two foraging stages are combined with the sine function to be nonlinear, which balances the ability of global searches and local explorations.
4. The proposed ITSO is utilized to optimize the learning rate, regularization coefficient, and node number of the BiLSTM layer, so that the diagnostic accuracy is significantly improved.

## 2. Fault Diagnosis Model Based on ITSO-CNN-BiLSTM

### 2.1. Introduction of Convolutional Neural Network

**Figure 1:**
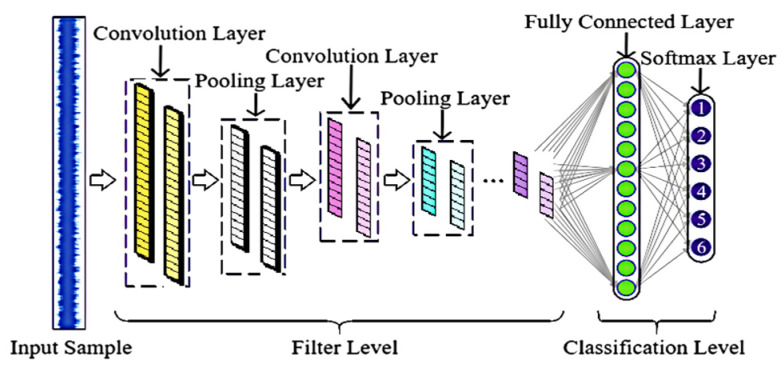
Schematic diagram of a one-dimensional CNN.

A convolutional neural network (CNN) is a typical feed-forward neural network, which usually contains five component modules: an input layer, convolutional layer, pooling layer, fully connected layer, and output layer. A schematic diagram of a one-dimensional CNN [21,22] is shown in Figure 1.

A CNN is characterized by sparse connectivity and weight sharing, which can reduce the number of parameters and decrease the complexity of the network, and then realize the feature extraction of the input layer. The formula for the operation of the convolutional layer is as follows:

(1) $$x_{i}^{out}=f\left(\sum_{i=1}^{n}\left(x_{i}^{in}\times w_{ij}\right)+b_{j}\right)$$

where $x_{i}^{out}$ denotes the output of the *i* th neuron; $x_{i}^{in}$ denotes the input of the *i* th neuron; $f\left(\cdot \right)$ is a nonlinear transformation aiming to enhance the nonlinearity of the network; $w_{ij}$ represents the weight value of the connection between $x_{i}^{in}$ and the *j* th neuron; *b_j_* indicates the *j* th offset of the output.

In this study, the maximum pooling method is adopted to reduce the error of the estimated mean shift due to the parameter error of the convolution layer, and the model is represented by the following function:

(2) $$\begin{array}{c} y_{i}^{out}=f_{max}\left(y_{q}^{in},y_{q+1}^{in}\right) \end{array}$$

where $y_{q}^{in}$ is the input of the *q* th neuron of the input feature surface; $f_{max}(\cdot )$ denotes the maximum value of the function; $y_{i}^{out}$ is the output value of the *i* th neuron of the output feature surface.

The fully connected layer is located at the end of the CNN. After feature extraction in the convolutional layer and dimensionality reduction in the pooling layer, the learned features are mapped to the labeled space of samples in the fully connected layer. The fully connected layer purifies the features and hands the output to the softmax layer for classification.

### 2.2. Introduction of BiLSTM Network

The LSTM network is an improvement of Recurrent Neural Networks (RNNs), which adds a new “forgetting mechanism”. In this mechanism, each cycle is weighted once to determine the influence of parameters from past data, so as to solve the problem of gradient explosion. Therefore, the running time of the network becomes shorter, and the accuracy becomes higher [23].

The function of the forgetting gate is to determine which information should be discarded or retained and complete the screening of memory content. The output of the forgetting gate is as follows:

(3) $$\begin{array}{c} f_{t}=\sigma \left(W_{xf}x_{t}+W_{hf}h_{t-1}+b_{f}\right) \end{array}$$

where *σ* denotes the sigmoid activation function with an output range of 0~1; $W_{xf}$ is the weight matrix between the input and the forgetting gate; $W_{hf}$ is the weight matrix between the historical output and the forgetting gate; $b_{f}$ is the forgetting gate bias term; $x_{t}$ is the current input state; $h_{t-1}$ is the previous moment output state; and $f_{t}$ is the output of the forgetting gate. The input gate is used to update the cell status, and the input gate output and candidate cell status are as follows:

(4) $$\begin{array}{c} i_{t}=\sigma \left(W_{xi}x_{t}+W_{hi}h_{t-1}+b_{i}\right) \end{array}$$

(5) $$\begin{array}{c} \hat{c_{t}}=tanh\left(W_{xc}x_{t}+W_{hc}h_{t-1}+b_{c}\right) \end{array}$$

where $i_{t}$ is the input gate output; $\hat{c_{t}}$ is the candidate cell state; $W_{xi}$ is the weight matrix between the input and input gates; $W_{hi}$ is the weight matrix between the historical output and the input gate; *tanh* is a function that adjusts the output of the network with a range of −1~1; $W_{xc}$ is the weight matrix between the input and cell states; $W_{hc}$ is the weight matrix between the historical outputs and the cell states; $b_{i}$ is the input gate bias; and $b_{c}$ is the cell state bias.

The current unit state $C_{t}$ is the sum of two parts; one is the information retention determined by the multiplication of the forgetting vector and the cell state of the previous layer, and the other part is the information update determined by the multiplication of the input gate and the current candidate state. The cell state is as follows:

(6) $$\begin{array}{c} C_{t}=f_{t}C_{t-1}+i_{t}\hat{c_{t}} \end{array}$$

The output gate is used to determine the next hidden state, and the output gate and unit outputs are as follows:

(7) $$\begin{array}{c} o_{t}=\sigma \left(W_{xo}x_{t}+W_{ho}h_{t-1}+b_{o}\right) \end{array}$$

(8) $$\begin{array}{c} h_{t}=o_{t}tanh\left(C_{t}\right) \end{array}$$

where $W_{xo}$ is the weight matrix between the input and output gates; $W_{ho}$ is the weight matrix between the historical output and the output gate; $b_{o}$ is the output gate bias; $o_{t}$ is the output of output gate; $h_{t}$ is the unit output.

The BiLSTM network is built, and the input time series data are input into the forward and reverse directions of the two LSTM networks for feature extraction, and the extracted features are linearly fused according to the corresponding weights as the final feature expression, which can improve the diagnostic accuracy of the model. [24,25].

### 2.3. Tuna Swarm Optimization Algorithm

Tuna swarm optimization (TSO) is a newly proposed fish swarm-based algorithm, which is mainly inspired by the cooperative foraging behavior of tuna swarms [26]. The algorithm simulates two foraging behaviors of tuna swarms: spiral foraging and parabolic foraging.

1. Initialization

The random initialization in the optimization space is shown as follows:

(9) $$\begin{array}{c} X_{i}^{int}=rand\left(ub-lb\right)+lb,i=1,2,\cdots ,NP \end{array}$$

where $X_{i}^{int}$ denotes the initial position of the *i* th individual, *ub* and *lb* denote the upper and lower limits of the search space, respectively, and *NP* indicates the total number of tuna populations.

2. Spiral foraging

Tuna groups chase prey by forming a tight spiral. In addition to chasing prey, tuna groups also exchange information with each other. Each tuna follows the previous one, so information can be shared between adjacent tuna. The expression of spiral foraging is as follows:

(10) $$\begin{array}{c} X_{i}^{t+1}=\left\{\begin{array}{c} \alpha_{1}\left(X_{best}^{t}+\beta \left|X_{best}^{t}-X_{i}^{t}\right|\right)+\alpha_{2}X_{i}^{t},i=1\\ \alpha_{1}\left(X_{best}^{t}+\beta \left|X_{best}^{t}-X_{i}^{t}\right|\right)+\alpha_{2}X_{i-1}^{t},i=2,3,\cdots ,NP \end{array}\right. \end{array}$$

(11) $$\begin{array}{c} \alpha_{1}=a+\left(1-a\right)\frac{t}{t_{max}} \end{array}$$

(12) $$\begin{array}{c} \alpha_{2}=\left(1-a\right)-\left(1-a\right)\frac{t}{t_{max}} \end{array}$$

(13) $$\begin{array}{c} \beta =e^{bl}cos\left(2\pi b\right) \end{array}$$

(14) $$\begin{array}{c} l=e^{3\cos\left(\left(\frac{t_{max}+1}{t}-1\right)\pi \right)} \end{array}$$

where $X_{i}^{t+1}$ denotes the position of the *i* th individual at *t* + 1 iterations; $X_{best}^{t}$ denotes the position of the current best individual; $\alpha_{1}$ indicates the weight coefficient of the current individual moving towards the best individual; $\alpha_{2}$ denotes the weight coefficient of the current individual moving to the previous individual; *t* denotes the number of current iterations; $t_{max}$ indicates the maximum number of iterations; constant *a* indicates the extent to which the tuna individual follows the position of the best individual and the previous individual in the initial stage; constant *b* is a random constant uniformly distributed between 0 and 1.

A random coordinate is considered to be generated in the search space as a reference point for spiral searching. It enables each individual to explore in a wider space and gives TSO a global exploration capability. The expression is as follows:

(15) $$\begin{array}{c} X_{i}^{t+1}=\left\{\begin{array}{c} \alpha_{1}\left(X_{rand}^{t}+\beta \left|X_{rand}^{t}-X_{i}^{t}\right|\right)+\alpha_{2}X_{i}^{t},\;i=1\\ \alpha_{1}\left(X_{rand}^{t}+\beta \left|X_{rand}^{t}-X_{i}^{t}\right|\right)+\alpha_{2}X_{i-1}^{t},i=2,3,\cdots ,NP \end{array}\right. \end{array}$$

where $X_{rand}^{t}$ indicates the randomly generated reference points in the optimization-seeking space.

3. Parabolic foraging

Tuna form a parabolic shape with food as a reference point and search around. Assuming that the selection probability of both methods is 50%, the two methods are performed simultaneously. The expression is as follows:

(16) $$\begin{array}{c} X_{i}^{t+1}=\left\{\begin{array}{c} X_{best}^{t}+rand\left(X_{best}^{t}-X_{i}^{t}\right)+TFP^{2}\left(X_{best}^{t}-X_{i}^{t}\right),\;if\;rand<0.5\\ TF\cdot P^{2}\cdot X_{i}^{t},ifrand>0.5 \end{array}\right. \end{array}$$

(17) $$\begin{array}{c} P={\left(1-\frac{t}{t_{max}}\right)}^{t/t_{max}} \end{array}$$

where *TF* denotes a random number that takes 1 or −1 randomly.

### 2.4. Improved Tuna Swarm Optimization Algorithm

1. Improvement of initialization

The improved mathematical model of Chebyshev chaotic mapping is shown in (18):

(18) $$\begin{array}{c} X_{n+1}=1-2(cos\left(2arccos\left(X_{n}\right)\right){)}^{2},\;X_{n}\in \left[0,1\right] \end{array}$$

The standard TSO optimization algorithm reduces the population diversity because the initial population is randomly generated. Therefore, improved Chebyshev chaotic mapping is used to initialize the tuna group to increase the diversity of the group. After the introduction of Chebyshev chaotic mapping, Formula (9) is rewritten as (19), as follows:

(19) $$X_{i}^{n}=X_{n+1}(Ub-Lb)+Lb,i=1,2,\cdots ,N$$

In the formula, *Ub* and *Lb* are the maximum and minimum boundary values of the independent variables. The initial population generated by Chebyshev chaotic mapping has more ergodic uniformity, which is helpful to improve the global search performance and search stability of the TSO algorithm.

2. Improvement of spiral foraging stage

According to Formulas (11) and (12), the two weight coefficients are linearly updated, which easily leads to the imbalance between the global search and the local exploration and prematurely falls into the local optimal solution. Therefore, the weight coefficients *α_1_* and *α_2_* are combined with the sine function to balance the global search and local exploration capabilities of the TSO algorithm. The new weight coefficient expression is shown in (20).

(20) $$\begin{array}{c} \begin{array}{c} \alpha_{1}^{\prime }=\alpha_{1Ori}+0.05sin\left(\frac{2\pi t}{T_{max}}\right)\\ \alpha_{2}^{\prime }=\alpha_{2Ori}-0.05sin\left(\frac{2\pi t}{T_{max}}\right) \end{array} \end{array}$$

In the formula, $\alpha_{1Ori}$ and $\alpha_{2Ori}$ represent two original weight functions, t is the current iteration value, and $T_{max}=550$ is the maximum number of iterations.

**Figure 2:**
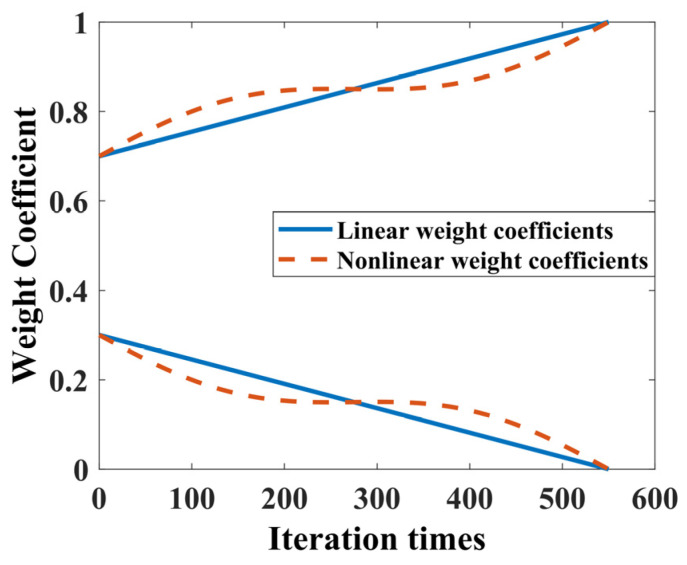
The improved weight coefficient compared with the change curve of the original weight coefficient.

The comparison between improved and original weight coefficients is shown in Figure 2. It can be seen that, under the nonlinear regulation, in the early iteration of the algorithm, the change amplitude of $\alpha_{1}^{\prime }$ and $\alpha_{2}^{\prime }$ can enhance the global search ability of the algorithm; in the late iteration, the values of $\alpha_{1}^{\prime }$ and $\alpha_{2}^{\prime }$ are gradually stabilized, so that the individuals in the populations can approach the optimal solution in the neighboring area and fully carry out local exploration, thus better balancing the global search and local exploration.

3. Improvement of parabolic foraging stage

In this paper, a new nonlinear weight coefficient *p* is proposed to improve the global search ability of TSO. The mathematical model is shown in (21) [27] as follows:

(21) $$\begin{array}{c} p_{1}=p_{init}-sin\left(\frac{\pi t}{2T_{max}}\right) \end{array}$$

where $p_{init}$ denotes the initial value of the original weight coefficient *p*.

**Figure 3:**
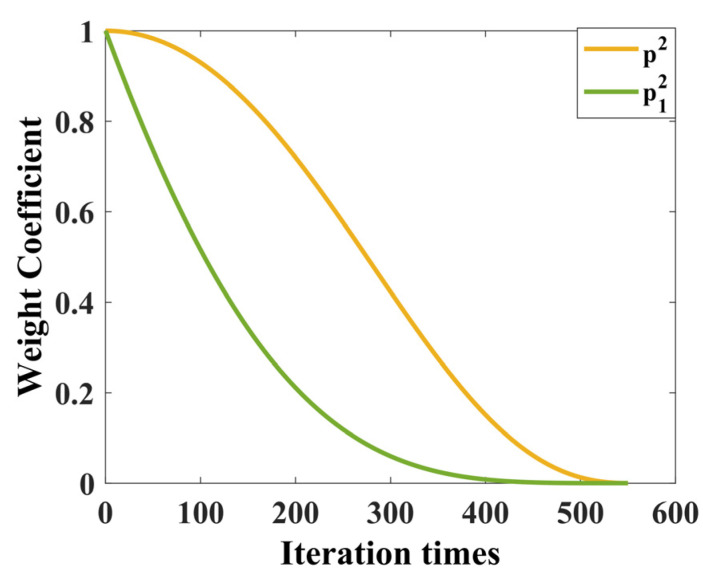
The improved weight parameter ${p_{1}}^{2}$ is compared with the original weight parameter $p^{2}$.

The improved weight parameter ${p_{1}}^{2}$ is compared with the original weight parameter $p^{2},$ and the results are shown in Figure 3. The improved weighting coefficient ${p_{1}}^{2}$ decreases rapidly in the early stage, and the tuna individuals can better follow the previous individual, which improves the global exploration ability.

### 2.5. Optimization Process of ITSO-CNN-BiLSTM

For ITSO-CNN-BiLSTM, the optimized parameters include the learning rate, regularization coefficient, and number of BiLSTM layer nodes. The optimization process is as follows:

(1) The data selected from different sensors are normalized and the training set and test set are divided.
(2) The pre-training parameters of CNN-BiLSTM are initialized, including the number of iterations, the learning rate, the momentum of learning rate, etc.
(3) The parameters of the ITSO algorithm are initialized, including the size of population, individual position, the maximum number of iterations, etc.
(4) Boundary settings are made for the three parameters to be optimized, namely, the learning rate, regularization coefficient, and number of nodes in the BiLSTM layer, and network training is carried out.
(5) Weight parameters $\alpha_{1}^{\prime }$ and $\alpha_{2}^{\prime }$ are updated, and $p_{1}$ is updated according to (20)~(21); a random quantity of *rand* ∈ [0, 1] is generated; if *rand* < 0.5, he spiral foraging phase is entered, and the position of the individual is updated according to (15); if *rand* ≥ 0.5, the parabolic foraging phase is entered, and the position of the individual is updated according to (16).

**Figure 4:**
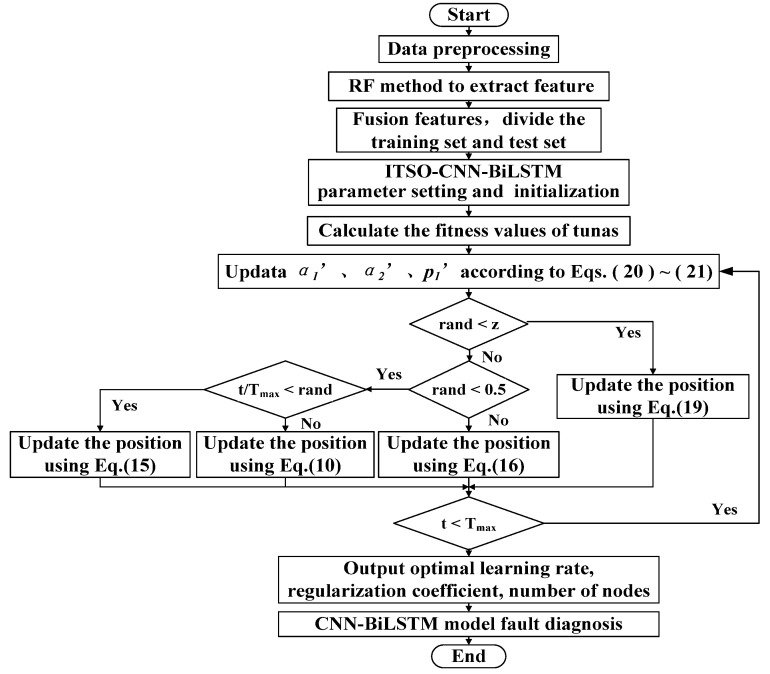
ITSO-CNN-BiLSTM diagnostic model flow chart.

During iterative optimization, the individual optimal value, group optimal value, and position vector of each individual are updated. The iteration is ended when the termination condition is reached, and the optimization results of the learning rate, the regularization coefficient, and the number of nodes in the BiLSTM layer are obtained. The flow scheme is shown in Figure 4.

## 3. Experimental Verification

**Figure 5:**
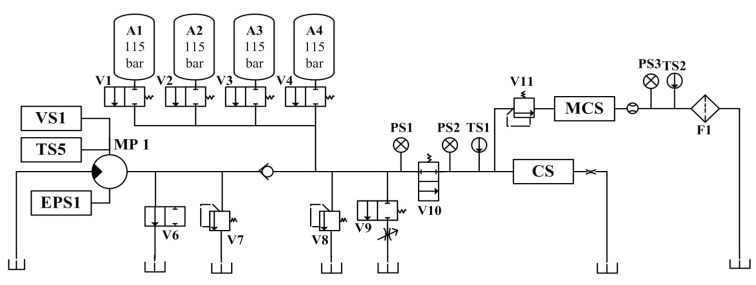
Structure of the main circuit of the test bench.

**Figure 6:**
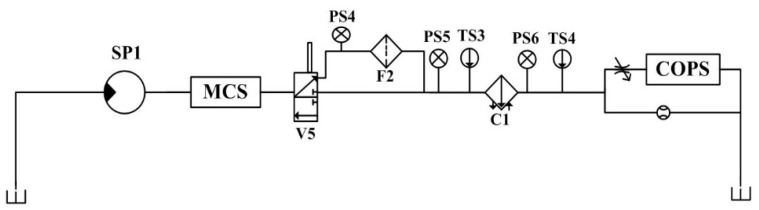
Structure of the secondary cooling and filtering circuit of the test bench.

The proposed method is validated using a hydraulic system dataset from the University of California, Irvine (UCI Center for Machine Learning and Intelligent Systems). The dataset is experimentally collected through a hydraulic test bench consisting of a main operating circuit (Figure 5) and a secondary cooling and filtering circuit (Figure 6) connected through a tank. There are six types of sensors (with a total number of fifteen) installed in the test bench. The system performs repeated constant load cycles with a 60 s cycle and records the signals collected by the sensors, including pressure (PS1–PS6), flow rate (FS1, FS2), temperature (TS1–TS4), motor power (EPS1), efficiency factor (SE), and vibration (VS1). The states of four hydraulic components (plunger pump, cooler, throttle, and accumulator) are quantitatively changed in the experiment to simulate the corresponding failure of each component, and the failure parameter settings of each hydraulic component are detailed in Table 1.

Pressure signals are collected by pressure sensors PS1~PS6 (sampling frequency 100 Hz), where each sensor contains 6000 attributes; flow signals are collected by flow sensors FS1 and FS2 (sampling frequency 10 Hz), where each sensor contains 600 attributes; motor power signals are collected by motor power sensor EPS1 (sampling frequency 100 Hz), where each sensor contains 6000 attributes; temperature signals are collected by temperature sensors TS1~TS4 (sampling frequency 1 Hz), where each sensor contains 60 attributes; motor efficiency coefficients are collected by efficiency coefficient sensor SE (sampling frequency 1 Hz), where each sensor contains 60 attributes; vibration signals are collected by vibration sensor VS1 (sampling frequency 1 Hz), where each sensor contains 60 attributes. The cooler contains three operating states: near total failure (732 instances), reduced efficiency (732 instances), and full efficiency (741 instances); valve states: optimal switching behavior (1125 instances), small lag (360 instances), severe lag (360 instances), and near total failure (360 instances); leaks in the pump: no leaks (1221 instances), weak leaks (492 instances), and severe leaks (492 instances); hydraulic accumulator operating conditions: optimal pressure (599 instances), slight depressurization (399 instances), severe depressurization (399 instances), and near total failure (808 instances).

When diagnosing the fault status of a hydraulic component, it is affected by the fault of other components, which increases the difficulty of diagnosis. For instance, when the plunger pump has minor internal leakage, the cooler has reduced cooling capacity, the throttle has degraded switching characteristics, and the accumulator has gas leakage; when the cooler is close to total failure, the plunger pump has internal leakage, the throttle has degraded switching characteristics, and the accumulator has gas leakage.

The ITSO-CNN-BiLSTM can be divided into an input layer, CNN network, BiLSTM network, and output layer. In the CNN network, there are two convolutional layers, one pooling layer, and one flatten layer in sequence. The BiLSTM network consists of two BiLSTM layers and a fully connected layer. The initial number of neurons in both BiLSTM layers is set to 32. The initial settings of the convolutional layers are as follows: the first convolutional layer adopts 16 × 1 convolutional kernels with a step size of 1; the second convolutional layer adopts 32 × 1 convolutional kernels with a step size of 1; the activation functions are all ReLU functions, and the filling methods are all chosen as ‘same’; the pooling layer chooses the maximum pooling method, and the size of the pooling kernels is initially set to 4 × 1. The number of output neurons in the softmax layer corresponds to different fault states of hydraulic components. The number of pre-training iterations is set to 550, and the pre-training learning rate is set to 0.01. Based on several experimental results, the degree of following the previous individual and the best individual in the initial stage is set as 0.7, the mutation probability is 0.05, and the population size is 30. In order to solve the problem that the random parameter setting has a great influence on the accuracy of the diagnostic model, the improved ITSO optimization algorithm is introduced to improve the accuracy of the CNN-BiLSTM network.

## 4. Experimental Results and Analysis

### 4.1. Experimental Verification and Analysis of Plunger Pump

Before identifying the three degrees of internal leakage (normal, minor internal leakage, and severe internal leakage) of the plunger pump, the random forest algorithm is utilized to compare the importance of the data collected by each sensor to realize the sensor selection. For each decision tree [28,29], the corresponding out-of-bag (OOB) data are first selected to calculate the OOB data error, which is recorded as errOOB1. Then, noise interference is randomly added to the OOB data, and the OOB data error is calculated again, which is recorded as errOOB2.

Assuming that there are *N* trees in the random forest, the importance of feature *X* is as follows:

(22) $$\begin{array}{c} X=\sum \frac{errOOB1-errOOB2}{N} \end{array}$$

**Figure 7:**
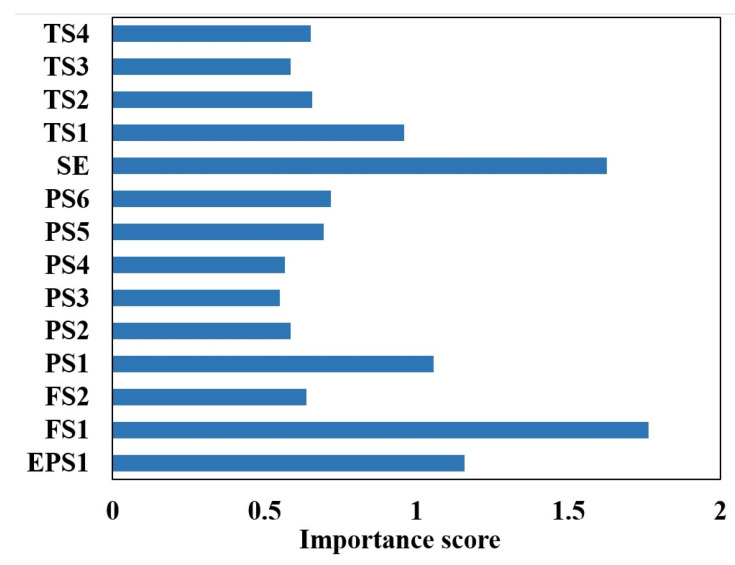
Sensor selection for plunger pump based on random forest.

The results of the calculations are shown in Figure 7.

Utilizing the feature importance index of the random forest model, the five aspects with the highest contribution to the internal leakage fault diagnosis of the plunger pump are selected, namely, motor power (EPS1), flow rate (FS1), pressure (PS1), motor efficiency coefficient (SE), and temperature (TS1). The sample properties are shown in Table 2.

1. Network parameters

The input of the network consists five sets of signals from EPS1, FS1, PS1, SE, and TS1, which are connected after normalization, and the width of the input is 12,720. After normalizing the data, the training set is set to 75% of the dataset, and the test set is set to 25% of the dataset.

2. Fault diagnosis results of plunger pump

**Figure 8:**
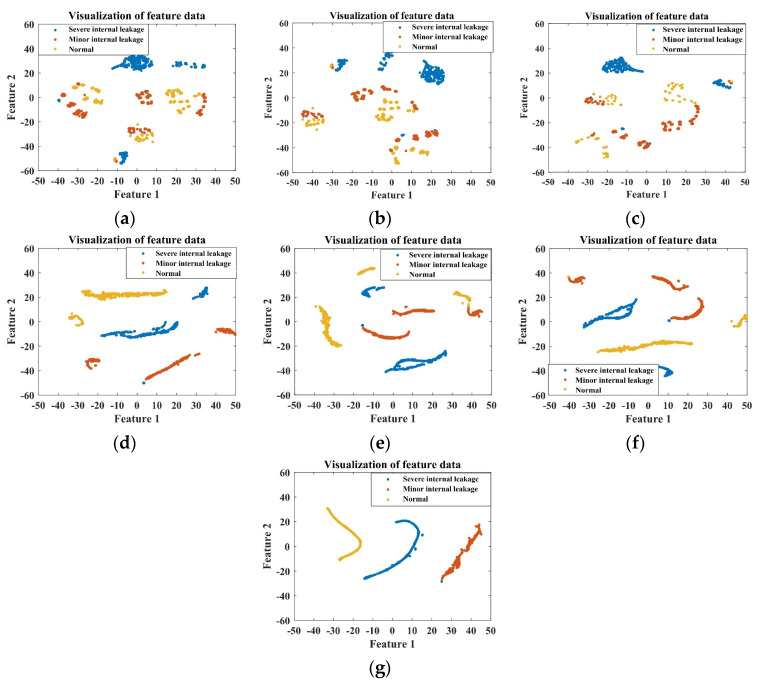
Visualization of the output of each layer of ITSO-CNN-BiLSTM network for plunger pump fault diagnosis with (**a**) input layer; (**b**) Conv_1; (**c**) Conv_2; (**d**) BiLSTM 1; (**e**) BiLSTM 2; (**f**) fully connected layer; (**g**) softmax layer.

The ITSO-CNN-BiLSTM network is utilized to fuse the data from the selected five sensors. For the training set, the ITSO-CNN-BiLSTM network achieves optimal performance at 538 iterations of training. As shown in Figure 8, the outputs of each layer are visualized by dimensionality reduction using the t-SNE technique.

It can be seen from Figure 8 that from the signals of the initial five sensors, it is difficult to distinguish the degree of internal leakage. As the features are extracted from each layer of the network, the separability among samples with different fault degrees gradually increases, and the final output features have the best clustering effect. This indicates that the ITSO-CNN-BiLSTM model has the ability to achieve the accurate classification of different internal leakage degrees.

In this paper, the recognition effectiveness of the proposed ITSO-CNN-BiLSTM model is analyzed using four commonly used metrics for evaluating the classification performance, namely, accuracy, precision, recall rate, and F1 score. *TP* is the number of samples of the target class that are correctly recognized, *TN* is the number of samples of other classes that are correctly recognized, *FP* is the number of samples of the target class that are incorrectly recognized, and *FN* is the number of samples of other classes that are incorrectly recognized. The evaluation indicators of the classification model are shown in (23)~(26) as follows:

(23) $$\begin{array}{c} Accuracy=\frac{TP+TN}{TP+FN+TN+FN} \end{array}\times 100\%$$

(24) $$\begin{array}{c} Precision=\frac{TP}{TP+FP} \end{array}\times 100\%$$

(25) $$\begin{array}{c} Recall=\frac{TP}{TP+FN}\times 100\% \end{array}$$

(26) $$\begin{array}{c} F1=\frac{2\times Recall\;Rate\times Precision}{Recall\;Rate+Precision} \end{array}$$

**Figure 9:**
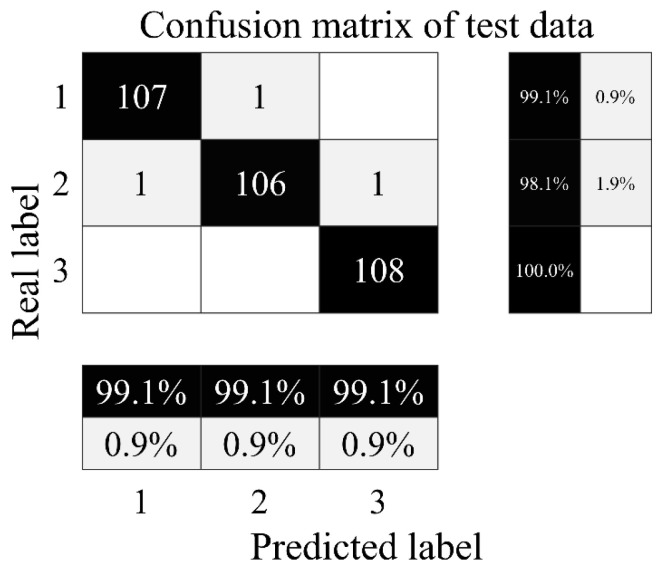
Confusion matrix of plunger pump test set. 1—Severe internal leakage; 2—minor internal leakage; 3—normal.

The confusion matrix of the plunger pump fault diagnosis test set is shown in Figure 9.

As can be seen in Figure 9, one of the severe internal leakage samples is misclassified as minor internal leakage, with accuracy of 99.07%; one of the minor internal leakage samples is misclassified as normal and one is misclassified as severe internal leakage, with accuracy of 98.15%; all normal samples are correctly identified, with accuracy of 100%; and the total accuracy of the test set is 99.07%.

In an effort to verify the advantages of the ITSO-CNN-BiLSTM model for plunger pump fault diagnosis, four models of CNN-LSTM, CNN-BiLSTM, TSO-CNN-BiLSTM, and ITSO-CNN-BiLSTM are utilized for fault diagnosis based on data of the abovementioned five sensors. The comparison of diagnostic results is shown in Table 3.

From the indicators of accuracy, precision, recall rate, and F1 score, it can be seen that the proposed ITSO-CNN-BiLSTM model has the best performance for plunger pump fault diagnosis compared with other models. Due to the adaptive optimization of parameters such as learning rate, regularization coefficient, and the number of BiLSTM layer nodes by improved TSO, the network structure reaches the optimal state, thus achieving the best diagnostic result.

The intra-class and inter-class distances corresponding to the three state samples are calculated using the outputs of each layer of the ITSO-CNN-BiLSTM network as a way to verify the classification ability of the proposed model.

$S_{B}^{k}$ indicates the intra-class variance of the *k*-dimensional sample and is calculated as follows:

(27) $$\begin{array}{c} S_{B}^{k}=\sum_{I=1}^{c}\frac{n_{i}}{n}{\left(m_{i}\left(k\right)-m\left(k\right)\right)}^{2} \end{array}$$

**Figure 10:**
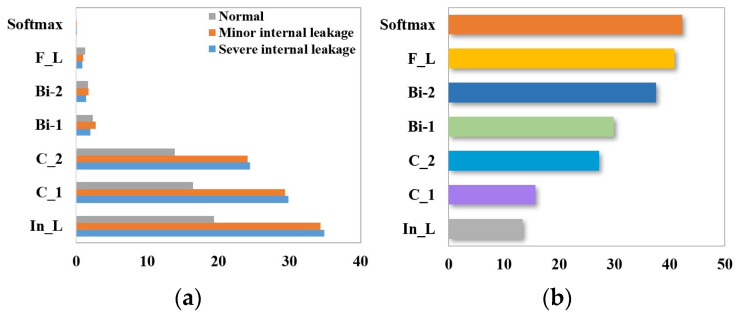
Intra-class and inter-class distances of samples with different fault degrees of plunger pump in each layer of ITSO-CNN-BiLSTM. (**a**) Intra-class distance; (**b**) inter-class distance.

$S_{W}^{k}$ represents the inter-class variance of the *k*-dimensional sample, calculated as follows:

(28) $$\begin{array}{c} S_{W}^{k}=\frac{1}{n}\sum_{i=1}^{c}\sum {\left(x\left(k\right)-m_{i}\left(k\right)\right)}^{2} \end{array}$$

where *n* denotes the total number of samples, $m_{i}\left(k\right)$ denotes the mean value of the sample $x\left(i\right)$ in the *k*-dimension, and $m\left(k\right)$ denotes the mean value of all samples in the *k*-dimension. The calculation results are shown in Figure 10.

As shown in Figure 10a,b, with the feature extraction in each layer, the intra-class distances of normal, minor internal leakage, and severe internal leakage samples decrease from 19.3546, 34.3678, and 34.8574 in the input layer to 0.0633, 0.0815, and 0.0686 in the softmax layer. The inter-class distance increases from 13.3033 in the input layer to 42.2237 in the softmax layer. From the inter-class distance and intra-class distance, it can be seen that the proposed method has the ability to significantly improve the separability of samples with different fault states.

After adding white noise with SNRs of 0 dB, 5 dB, and 10 dB, the ITSO-CNN-BiLSTM network classifies the fault degrees of the plunger pump with accuracies of 89.2%, 93.83%, and 97.53%, respectively. The results are shown in Table 4.

From the indicators of accuracy, precision, recall rate, and F1 score, it can be seen that although the classification ability for noise-added data is weaker than that of the original data, ITSO-CNN-BiLSTM still achieves accurate fault diagnosis with good robustness to noise.

### 4.2. Experimental Verification and Analysis of Cooler

**Figure 11:**
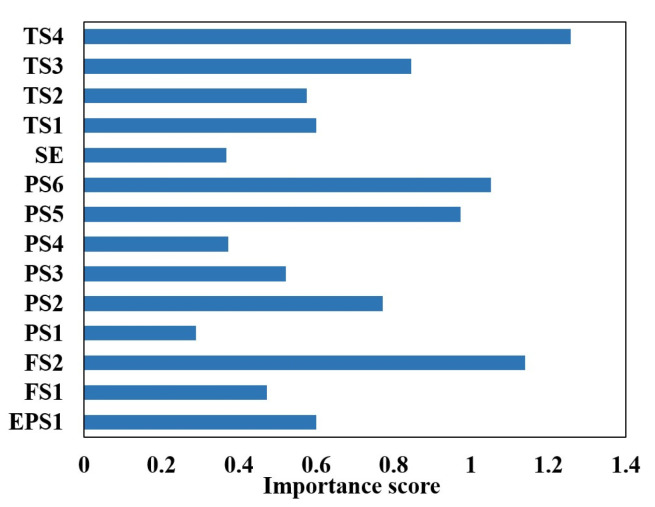
Sensor selection for cooler failure based on random forest.

The identification of the three degrees of cooling capacity degradation (close to total failure, reduced efficiency, and full efficiency) is preceded by sensor selection using the random forest algorithm. The calculation equations are shown in (22), and the results are shown in Figure 11.

RF feature selection can better capture the nonlinear relationship between cooler faults and sensor features and can consider the causality between high-dimensional feature data, namely, flow rate (FS2), pressure (PS5 and PS6), and temperature (TS3 and TS4). The sample properties are shown in Table 5.

1. Network parameters

The process of ITSO-CNN-BiLSTM is the same as the fault diagnosis process of the aforementioned plunger pump, and the network parameters are consistent. The input of the network consists of five sets of normalized signals from FS2, PS5, PS6, TS3, and TS4. The width of the input is 12,720. The softmax-layer output neuron number is three, as shown in Table 5, which corresponds to the three degrees of cooling capacity degradation.

2. Fault diagnosis results of cooler

**Figure 12:**
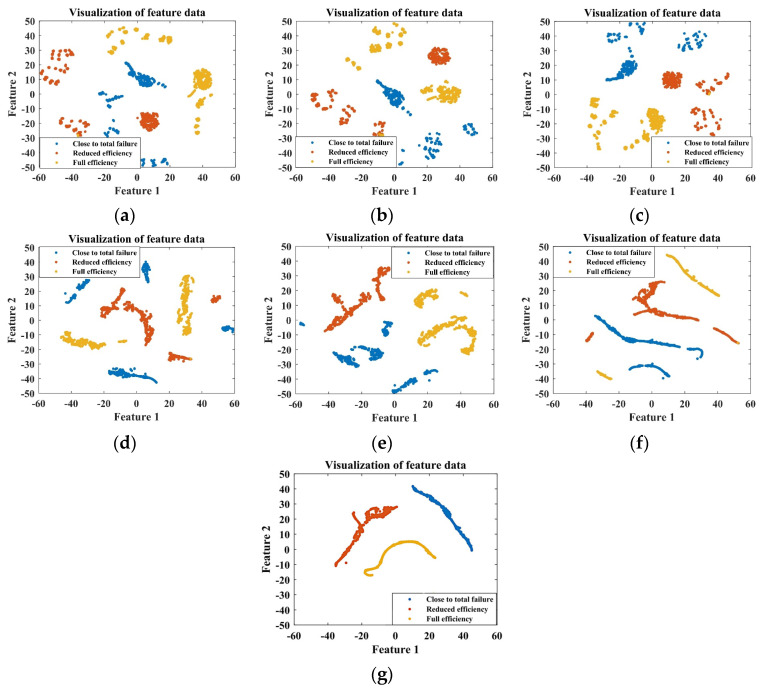
Visualization of the outputs of each layer of the ITSO-CNN-BiLSTM network for cooler fault diagnosis with (**a**) input layer; (**b**) Conv_1; (**c**) Conv_2; (**d**) BiLSTM 1; (**e**) BiLSTM 2; (**f**) fully connected layer; (**g**) softmax layer.

For the training set, the ITSO-CNN-BiLSTM network achieves optimal performance at 514 iterations of training. As shown in Figure 12, the outputs of each layer are visualized using the t-SNE technique.

It can be seen from Figure 12 that from the signals of the initial five sensors, it is difficult to distinguish the degrees of cooling capacity degradation. As the features are extracted from each layer of the network, the clustering effect of the samples is gradually improved. For the final output features, there is a clear distinction among the three fault states. This shows that the ITSO-CNN-BiLSTM model has the ability to achieve the accurate classification of different degrees of cooling capacity degradation.

**Figure 13:**
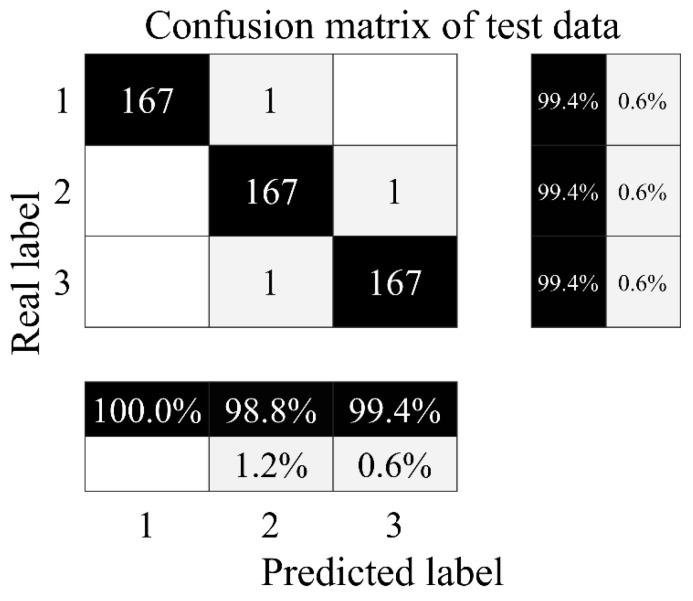
Confusion matrix of cooler test set. 1—Close to total failure; 2—reduced efficiency; 3—full efficiency.

As shown in Figure 13, one of the close-to-total-failure samples is misclassified as reduced efficiency, with accuracy of 99.4%; one reduced-efficiency sample is misclassified as full efficiency, with accuracy of 99.4%; one full-efficiency sample is misclassified as reduced efficiency, with accuracy of 99.4%; and the total accuracy of the test set is 99.4%.

In order to verify the excellence of the proposed model, four models, CNN-LSTM, CNN-BiLSTM, TSO-CNN-LSTM, and ITSO-CNN-BiLSTM, are used to diagnose the faults of the cooler. The comparison of diagnostic results is shown in Table 6.

From the indicators of accuracy, precision, recall rate, and F1 score, it can be seen that the proposed ITSO-CNN-BiLSTM model has the best performance for cooler fault diagnosis compared with other models.

**Figure 14:**
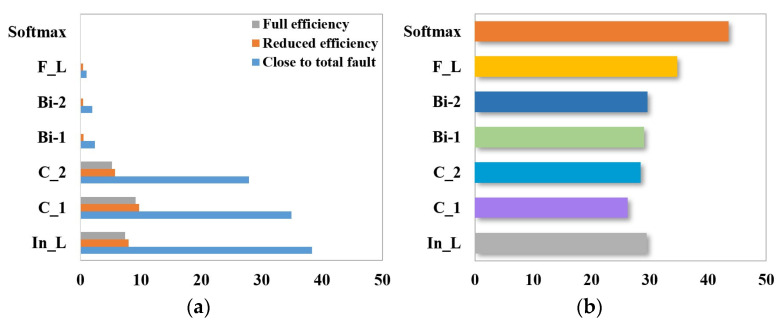
Intra-class and inter-class distances of samples with different fault degrees of cooler at each layer of ITSO-CNN-BiLSTM. (**a**) Intra-class distance; (**b**) inter-class distance.

The intra-class and inter-class distances corresponding to the three state samples are calculated using the outputs of each layer of ITSO-CNN-BiLSTM, as shown in Figure 14.

As shown Figure 14, the intra-class distance of the close-to-total-failure samples decreases from 38.3812 in the input layer to 0.0035 in the softmax layer, and correspondingly, the intra-class distances of the reduced-efficiency and full-efficiency samples decrease from 7.9533 and 7.4173 to 0.0053 and 0.0026, respectively. The inter-class distance increases from 29.4588 in the input layer to 43.4923 in the softmax layer. As the feature extraction is performed in each layer, the inter-class distance gradually increases and the intra-class distance gradually decreases, and the separability of the three types of samples gradually increases.

After adding white noise with SNRs of 0 dB, 5 dB, and 10 dB, the ITSO-CNN-BiLSTM network classifies the degree of cooling capacity degradation with accuracies of 89.88%, 94.05%, and 97.62%, respectively. The results are shown in Table 7.

From the indicators of accuracy, precision, recall rate, and F1 score, it can be seen that although the classification ability for noise-added data is weaker than that of the original data, ITSO-CNN-BiLSTM can still achieve accurate cooler fault diagnosis with good robustness to noise.

### 4.3. Experimental Verification and Analysis of Throttle Valve

**Figure 15:**
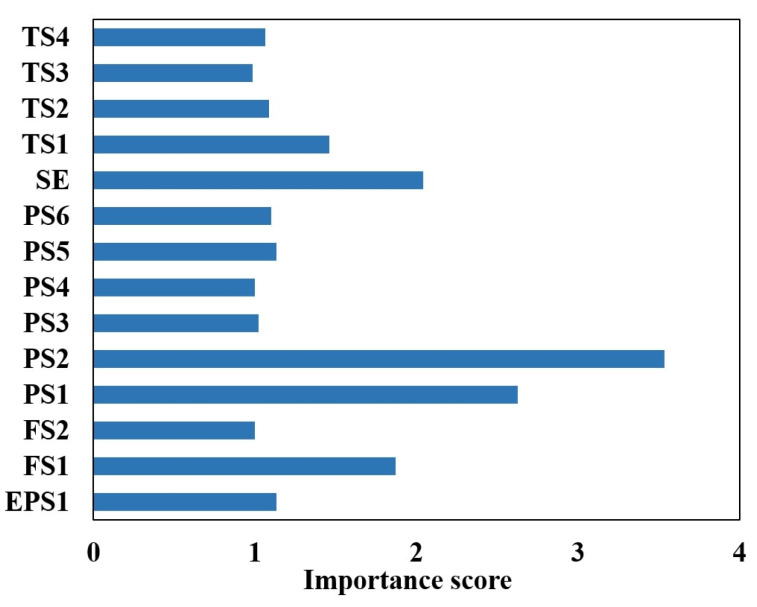
Sensor selection for throttle valve failure based on random forest.

The identification of the four degrees of throttle switching characteristics’ degradation (near-complete failure, severe hysteresis, minor hysteresis, and optimal switching) is preceded by sensor selection using the random forest algorithm. The calculation equations are shown in (22), and the results are shown in Figure 15.

The total number of sensors used to detect the state of the hydraulic system is 14, and not all of them are directly related to the throttle valve. RF calculates the importance of each sensor feature and selects the sensor features ranked in the top 5 for throttle valve troubleshooting, i.e., flow (FS1), pressure (PS1 and PS2), motor efficiency coefficient (SE), and temperature (TS1). The sample properties are shown in Table 8.

1. Network parameters

The process of ITSO-CNN-BiLSTM is the same as the fault diagnosis process of the aforementioned plunger pump, and the network parameters are consistent. The input of the network consists of five sets of normalized signals from FS1, PS1, PS2, SE, and TS1. The width of the input signal is 12,720. The softmax-layer output neuron number is four, as shown in Table 8, which corresponds to the four degrees of degradation of the throttle switching characteristics.

2. Fault diagnosis results of throttle valve

**Figure 16:**
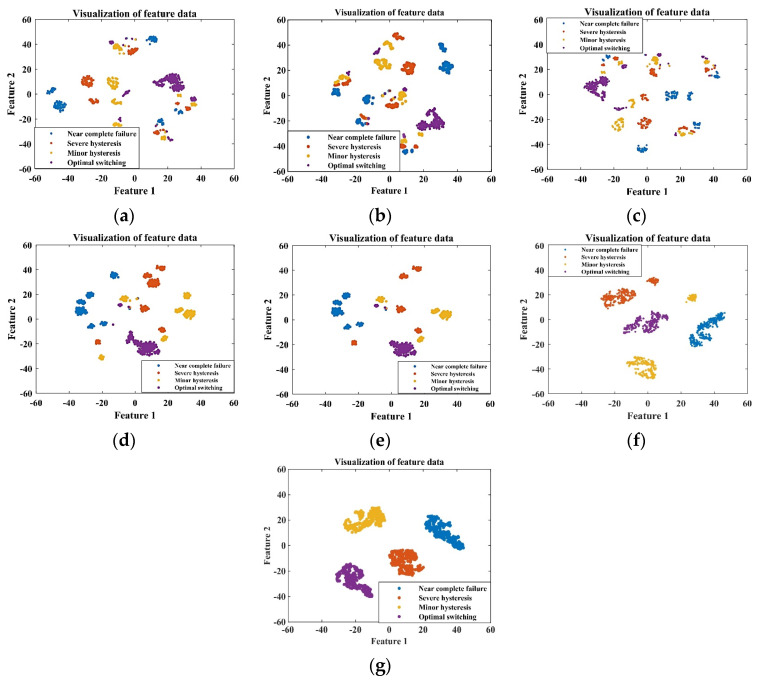
Visualization of the outputs of each layer of the ITSO-CNN-BiLSTM network for throttle valve fault diagnosis with (**a**) input layer; (**b**) Conv_1; (**c**) Conv_2; (**d**) BiLSTM 1; (**e**) BiLSTM 2; (**f**) fully connected layer; (**g**) softmax layer.

The ITSO-CNN-BiLSTM network is used to fuse the data from the selected five sensors. For the training set, the ITSO-CNN-BiLSTM network achieves optimal performance after 504 iterations of training. The outputs of each layer are visualized as shown in Figure 16.

It can be seen from Figure 16 that from the data of the initial five sensors, it is difficult to distinguish the degrees of throttle fault. As the features are extracted from each layer of the network, the separability among samples with different fault degrees gradually increases, and the final output features have the best clustering effect. This indicates that the ITSO-CNN-BiLSTM model has the ability to achieve the accurate classification of throttle fault degrees.

**Figure 17:**
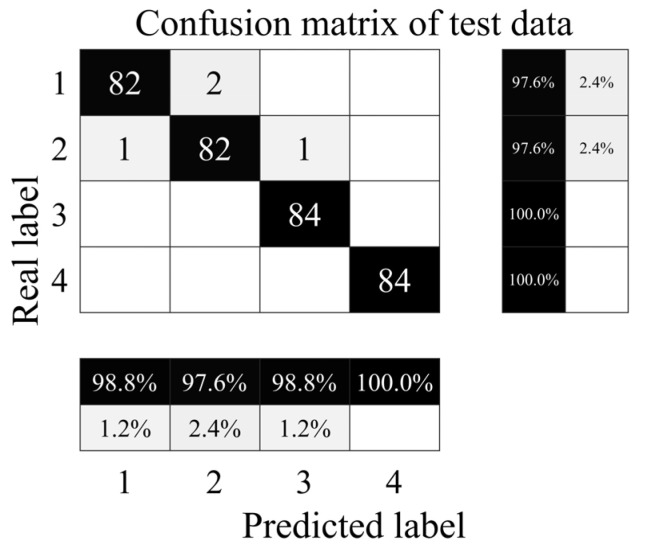
Confusion matrix of throttle valve test set. 1—Near-complete failure; 2—severe hysteresis; 3—minor hysteresis; 4—optimal switching.

The confusion matrix for the throttle fault diagnosis test set is shown in Figure 17.

As shown in Figure 17, all minor hysteresis and optimal switching samples are correctly diagnosed, with accuracy of 100%; two near-complete failure samples are misclassified as severe hysteresis, with accuracy of 97.6%; one severe hysteresis sample is misclassified as near-complete failure, and one severe hysteresis sample is misclassified as minor hysteresis, with accuracy of 97.6%; and the total accuracy of the test set is 98.81%. The results show that the proposed method has the ability to achieve the accurate identification of the degradation degree of throttle switching characteristics.

To verify the advantages of the ITSO-CNN-BiLSTM model for throttle fault diagnosis, CNN-LSTM, CNN-BiLSTM, TSO-CNN-LSTM, and ITSO-CNN-BiLSTM are utilized for fault diagnosis based on the data of the five abovementioned sensors, and the results are shown in Table 9.

From the indicators of accuracy, precision, recall rate, and F1 score, it can be seen that the proposed ITSO-CNN-BiLSTM model has the best performance for throttle valve fault diagnosis compared to other models.

**Figure 18:**
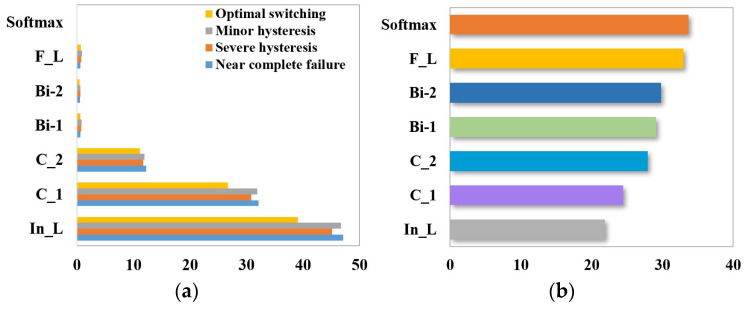
Intra-class and inter-class distances of samples with different fault degrees of throttle valve in each layer of ITSO-CNN-BiLSTM. (**a**) Intra-class distance; (**b**) inter-class distance.

As shown in Figure 18, the intra-class and inter-class distances corresponding to the four state samples are calculated using the outputs of each layer of the ITSO-CNN-BiLSTM network.

As shown in Figure 18, the intra-class distances of near-complete failure, severe hysteresis, minor hysteresis, and optimal switching samples decrease from 47.1109, 45.1478, 46.7275, and 39.1057 in the input layer to 0.0005, 0.0013, 0.0022, and 0.0011 in the softmax layer, respectively. The inter-class distance increases from 21.8299 in the input layer to 33.6469 in the softmax layer. As the intra-class distance gradually decreases and the inter-class distance gradually increases, the separability of the samples increases.

After adding white noise with SNRs of 0 dB, 5 dB, and 10 dB, the ITSO-CNN-BiLSTM network classifies the degradation degree of throttle switching characteristics with accuracies of 88.01%, 91.67%, and 96.73%, respectively. The results are shown in Table 10.

From the indicators of accuracy, precision, recall rate, and F1 score, it can be seen that although the classification ability for noise-added data is weaker than that of the original data, ITSO-CNN-BiLSTM still achieves accurate throttle fault diagnosis with good robustness to noise.

### 4.4. Experimental Verification and Analysis of Accumulator

**Figure 19:**
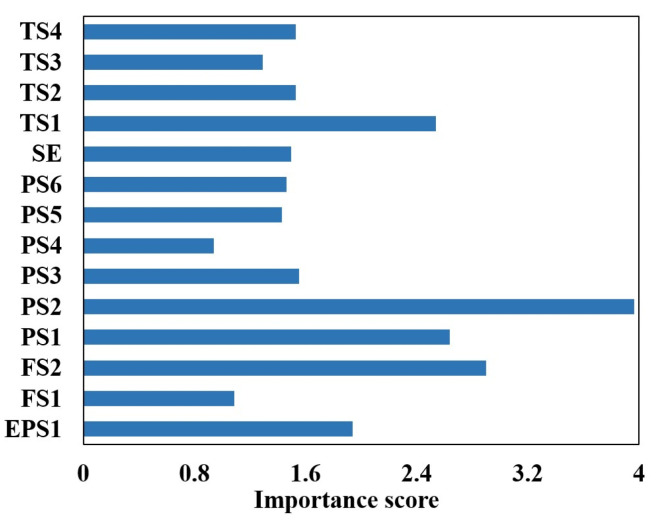
Sensor selection for accumulator based on random forest.

The identification of the four degrees of accumulator gas leakage (near-complete failure, severe depressurization, minor depressurization, and optimal pressure) is preceded by sensor selection using the random forest algorithm. The calculation equations are shown in (22), and the results are shown in Figure 19.

Signals from the top five sensors ranked in importance in Figure 19 are selected to identify the four degrees of gas leakage within the accumulator, namely, motor power (EPS1), flow rate (FS2), pressure (PS1 and PS2), and temperature (TS1). The sample properties are shown in Table 11.

1. Network parameters

The process of ITSO-CNN-BiLSTM is the same as the fault diagnosis process of the aforementioned plunger pump, and the network parameters are consistent. The input of the network consists five sets of normalized signals from EPS1, FS2, PS1, PS2, and TS1. The width of the input signal is 18,660. The softmax-layer output neuron number is four, as shown in Table 11, which corresponds to the four degrees of accumulator gas leakage.

2. Fault diagnosis results of accumulator

**Figure 20:**
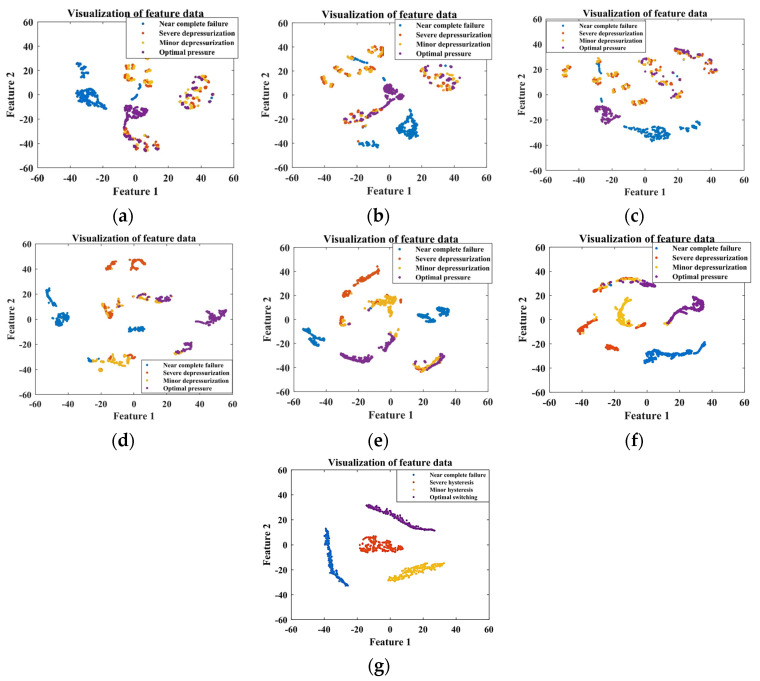
Visualization of the outputs of each layer of the ITSO-CNN-BiLSTM network for accumulator fault diagnosis with (**a**) input layer; (**b**) Conv_1; (**c**) Conv_2; (**d**) BiLSTM 1; (**e**) BiLSTM 2; (**f**) fully connected layer; (**g**) softmax layer.

The ITSO-CNN-BiLSTM network is utilized to fuse the data from the selected five sensors. For the training set, the ITSO-CNN-BiLSTM network achieves optimal performance after 560 iterations of training. The outputs of each layer are visualized using the t-SNE technique, as shown in Figure 20.

As shown in Figure 20, from the data of the initial five sensors, it is difficult to distinguish the degrees of accumulator gas leakage. As the features are extracted from each layer of the network, the separability among samples with different fault degrees gradually increases, and the final output features have the best clustering effect. This indicates that the ITSO-CNN-BiLSTM model has the ability to achieve the accurate classification of accumulator gas leakage degrees.

**Figure 21:**
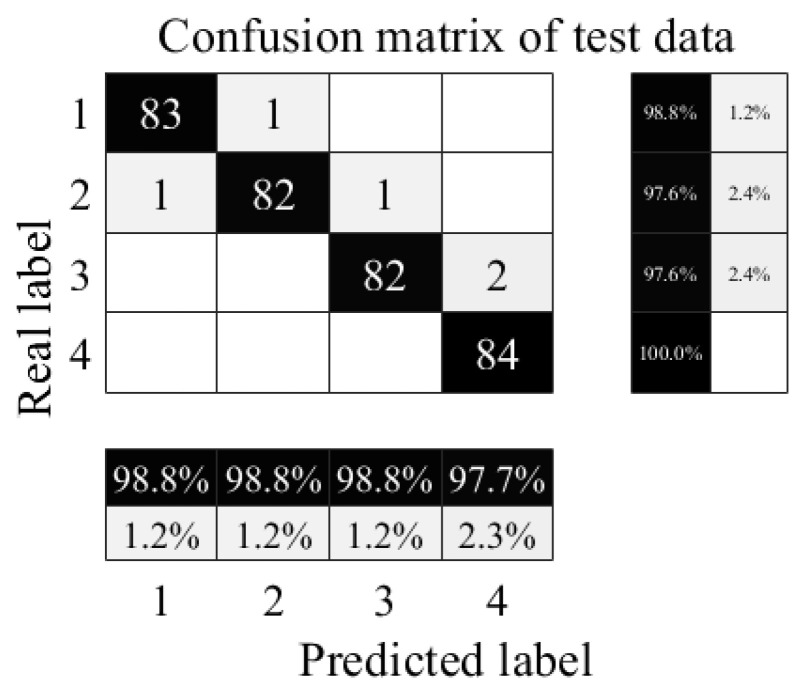
Confusion matrix of accumulator test set. 1—Near-complete failure; 2—severe depressurization; 3—minor depressurization; 4—optimal pressure.

The confusion matrix of the accumulator fault diagnosis test set is shown in Figure 21.

It can be seen in Figure 21 that all samples of optimal pressure are correctly diagnosed, with accuracy of 100%; one sample of near-complete failure is misclassified as severe depressurization, with accuracy of 98.8%; one sample of severe depressurization is misclassified as near-complete failure and one sample of severe depressurization is misclassified as minor depressurization, with accuracy of 97.6%; two minor depressurization samples are misclassified as optimal pressure, with accuracy of 97.6%; and the total accuracy of the test set is 98.51%. The results show that the proposed method achieves the accurate identification of the gas leakage degree of the accumulator.

In order to verify the advantages of ITSO-CNN-BiLSTM for accumulator fault diagnosis, CNN-LSTM, CNN-BiLSTM, TSO-CNN-LSTM, and ITSO-CNN-BiLSTM are utilized for fault diagnosis based on data of the five abovementioned sensors. The results are shown in Table 12.

From the indicators of accuracy, precision, recall rate, and F1 score, it can be seen that the proposed ITSO-CNN-BiLSTM model has the best performance for accumulator fault diagnosis compared to other models.

**Figure 22:**
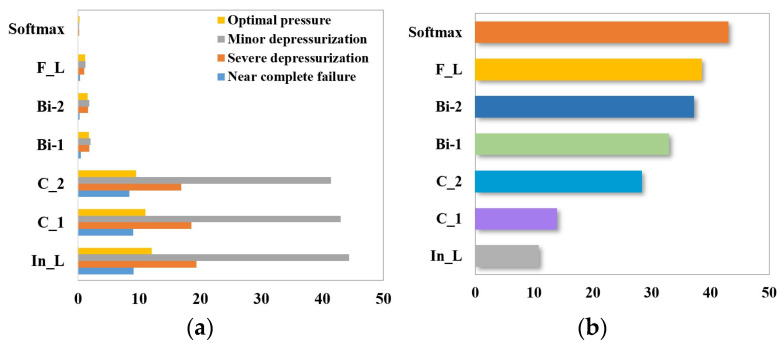
Intra-class and inter-class distances of samples with different fault degrees of accumulator at each layer of ITSO-CNN-BiLSTM. (**a**) Intra-class distance; (**b**) inter-class distance.

The intra-class and inter-class distances corresponding to the four state samples are calculated using the outputs of each layer of the ITSO-CNN-BiLSTM network, and the results are shown in Figure 22.

As shown in Figure 22, the intra-class distance of the severe depressurization samples decreases from 19.3749 in the input layer to 0.1487 in the softmax layer as feature extraction is performed in each layer of the network, and correspondingly, the intra-class distances of near-complete failure, minor depressurization, and optimal pressure samples decrease from 9.0962, 44.4011, and 12.0501 in the input layer to 0.0572, 0.1487, 0.1575, and 0.2251 in the softmax layer. The inter-class distances increase from 10.7861 in the input layer to 43.0283 in the softmax layer. As the intra-class distances decrease and the inter-class distances increase, the separability of the samples gradually increases.

After adding white noise with SNRs of 0 dB, 5 dB, and 10 dB, the ITSO-CNN-BiLSTM network identifies the degrees of gas leakage of the accumulator with accuracies of 87.5%, 91.07%, and 96.13%, respectively. The results are shown in Table 13.

From the indicators of accuracy, precision, recall rate, and F1 score, it can be seen that although the classification ability for noise-added data is weaker than that of the original data, ITSO-CNN-BiLSTM still achieves the accurate fault diagnosis of the accumulator with good robustness to noise.

In reference [2], the author proposes a multi-channel model with a CNN as the core, which has fault diagnosis accuracy of 98.98%, 100%, 100%, and 99.35% for the four components of plunger pump, cooler, throttle valve, and accumulator, respectively. However, it should be noted that the sample distribution in the two papers is different. In reference [2], 80% of the data are divided into the training set and the remaining 20% are divided into the testing set. In this article, the proportion of training samples is 75%, and the proportion of testing samples is 25%. The proportion of training samples in the total sample is smaller than reference [2]. It can be seen that the proposed model is tested under a more stringent condition.

The configuration of the computer utilized in our research is as follows: Intel Core i3-10100 CPU, 8 GB RAM, and Matlab 2019b. The offline training time for the four components is 799 s (plunger pump), 765 s (cooler), 824 s (throttle valve), and 781 s (accumulator). The diagnostic time for the four components is 145 s (plunger pump), 120 s (cooler), 177 s (throttle valve), and 163 s (accumulator). It can be seen that the proposed model has satisfactory computational efficiency.

## 5. Conclusions

In this paper, a multi-sensor information fusion model using ITSO-CNN-BiLSTM is proposed to realize the fault diagnosis of four components: plunger pump, cooler, throttle valve, and accumulator. The random forest algorithm is utilized to carry out sensor selection from six types of sensors. Based on the fusion feature extraction of the CNN and the comprehensive utilization of forward and backward features in BiLSTM, the effective diagnosis of hydraulic component fault status is achieved. The ITSO algorithm is adopted to adaptively optimize the learning rate, regularization coefficient, and node number of BiLSTM network. Improved Chebyshev chaotic mapping and the nonlinear reduction strategy are adopted to improve population initialization and individual position updating strategies, further promoting the optimization effect of TSO. The experimental results show that the proposed ITSO-CNN-BiLSTM network is an efficient fault diagnosis model for hydraulic components. The diagnostic results of noisy signals with SNR of 0 dB, 5 dB, and 10 dB show that the proposed method has good noise robustness. The research provides a reasonable and effective method for the intelligent fault diagnosis of hydraulic system.

## Figures and Tables

**Table 1:** Fault parameter settings of hydraulic components.

Hydraulic Component	Running State	Control Parameter	Limitation
Cooler (C1)	Cooling capacity decreased	Duty ratio of fan	0~100%
Throttle valve (V10)	Switch characteristic degradation	Control current	0~100% (rated)
Plunger pump (MP1)	Internal leakage	Adjusting hole	0.2/0.25 mm
Accumulator (A1–A4)	Gas leakage	Precharge pressure	9/10/11/11.5 MPa

**Table 2:** Sample properties of plunger pump.

Plunger Pump Status	Number of Training Samples	Number of Test Samples	Failure Tags
Severe internal leakage	324	108	001
Minor internal leakage	324	108	010
Normal	324	108	011

**Table 3:** Evaluation indicators for plunger pump.

Model	Accuracy	Precision	Recall	F1 Score
CNN-LSTM	0.9290	0.9296	0.9290	0.9292
CNN-BiLSTM	0.9537	0.9558	0.9537	0.9541
TSO-CNN-BiLSTM	0.9722	0.9722	0.9722	0.9721
ITSO-CNN-BiLSTM	0.9907	0.9907	0.9907	0.9907

**Table 4:** Evaluation indicators for plunger pump after noise addition.

SNR	Accuracy	Precision	Recall	F1 Score
0 dB	0.8920	0.8944	0.8920	0.8926
5 dB	0.9383	0.9383	0.9383	0.9383
10 dB	0.9753	0.9752	0.9753	0.9752

**Table 5:** Sample properties of cooler.

Cooler Status	Number of Training Samples	Number of Test Samples	Failure Tags
Close to total failure	504	168	001
Reduced efficiency	504	168	010
Full efficiency	504	168	011

**Table 6:** Evaluation indicators for cooler.

Model	Accuracy	Precision	Recall	F1 Score
CNN-LSTM	0.9385	0.9392	0.9385	0.9387
CNN-BiLSTM	0.9643	0.9643	0.9643	0.9643
TSO-CNN-BiLSTM	0.9821	0.9821	0.9821	0.9821
ITSO-CNN-BiLSTM	0.9940	0.9941	0.9940	0.9941

**Table 7:** Evaluation indicators for cooler after noise addition.

SNR	Accuracy	Precision	Recall	F1 Score
0 dB	0.8988	0.9039	0.8988	0.8997
5 dB	0.9405	0.9428	0.9405	0.9408
10 dB	0.9762	0.9768	0.9762	0.9763

**Table 8:** Sample properties of throttle valve.

Throttle Valve Status	Number of Training Samples	Number of Test Samples	Failure Tags
Near-complete failure	252	84	001
Severe hysteresis	252	84	010
Minor hysteresis	252	84	011
Optimal switching	252	84	100

**Table 9:** Evaluation indicators for throttle valve.

Model	Accuracy	Precision	Recall	F1 Score
CNN-LSTM	0.9107	0.9125	0.9107	0.9109
CNN-BiLSTM	0.9464	0.9469	0.9464	0.9464
TSO-CNN-BiLSTM	0.9702	0.9704	0.9702	0.9702
ITSO-CNN-BiLSTM	0.9881	0.9881	0.9881	0.9881

**Table 10:** Evaluation indicators for throttle valve after noise addition.

SNR	Accuracy	Precision	Recall	F1 Score
0 dB	0.8801	0.8814	0.8810	0.8807
5 dB	0.9167	0.9164	0.9167	0.9163
10 dB	0.9673	0.9672	0.9673	0.9670

**Table 11:** Sample properties of accumulator.

Accumulator Status	Number of Training Samples	Number of Test Samples	Failure Tags
Near-complete failure	252	84	001
Severe depressurization	252	84	010
Minor depressurization	252	84	011
Optimal pressure	252	84	100

**Table 12:** Evaluation indicators for accumulator.

Model	Accuracy	Precision	Recall	F1 Score
CNN-LSTM	0.9048	0.9054	0.9048	0.9049
CNN-BiLSTM	0.9435	0.9447	0.9435	0.9429
TSO-CNN-BiLSTM	0.9643	0.9643	0.9643	0.9643
ITSO-CNN-BiLSTM	0.9851	0.9852	0.9851	0.9851

**Table 13:** Evaluation indicators for accumulator after noise addition.

SNR	Accuracy	Precision	Recall	F1 Score
0 dB	0.8750	0.8757	0.8750	0.8750
5 dB	0.9107	0.9111	0.9107	0.9106
10 dB	0.9613	0.9617	0.9613	0.9612

## Data Availability

Data are contained within the article.
